# Abnormal morphology and function in retinal ganglion cells derived from patients-specific iPSCs generated from individuals with Leber’s hereditary optic neuropathy

**DOI:** 10.1093/hmg/ddac190

**Published:** 2022-08-10

**Authors:** Zhipeng Nie, Chenghui Wang, Jiarong Chen, Yanchun Ji, Hongxing Zhang, Fuxin Zhao, Xiangtian Zhou, Min-Xin Guan

**Affiliations:** Division of Medical Genetics and Genomics, The Children’s Hospital, Zhejiang University School of Medicine and National Clinical Research Center for Child Health, Hangzhou, Zhejiang, China; Institute of Genetics and Department of Human Genetics, Zhejiang University School of Medicine, Hangzhou, Zhejiang, China; Institute of Genetics and Department of Human Genetics, Zhejiang University School of Medicine, Hangzhou, Zhejiang, China; Division of Medical Genetics and Genomics, The Children’s Hospital, Zhejiang University School of Medicine and National Clinical Research Center for Child Health, Hangzhou, Zhejiang, China; Institute of Genetics and Department of Human Genetics, Zhejiang University School of Medicine, Hangzhou, Zhejiang, China; Division of Medical Genetics and Genomics, The Children’s Hospital, Zhejiang University School of Medicine and National Clinical Research Center for Child Health, Hangzhou, Zhejiang, China; Institute of Genetics and Department of Human Genetics, Zhejiang University School of Medicine, Hangzhou, Zhejiang, China; Department of Ophthalmology, The First Affiliated Hospital, Shandong First Medical University, Jinan, Shandong, China; School of Optometry and Ophthalmology and Eye Hospital, Wenzhou Medical University, Wenzhou, Zhejiang, China; School of Optometry and Ophthalmology and Eye Hospital, Wenzhou Medical University, Wenzhou, Zhejiang, China; Division of Medical Genetics and Genomics, The Children’s Hospital, Zhejiang University School of Medicine and National Clinical Research Center for Child Health, Hangzhou, Zhejiang, China; Institute of Genetics and Department of Human Genetics, Zhejiang University School of Medicine, Hangzhou, Zhejiang, China; Department of Ophthalmology, The First Affiliated Hospital, Shandong First Medical University, Jinan, Shandong, China; Zhejiang Provincial Key Lab of Genetic and Developmental Disorder, Hangzhou, Zhejiang, China; Joint Institute of Genetics and Genomic Medicine Between Zhejiang University and University of Toronto, Zhejiang University, Hangzhou, Zhejiang, China

## Abstract

Leber’s hereditary optic neuropathy (LHON) is a maternally inherited eye disease that results from degeneration of retinal ganglion cells (RGC). Mitochondrial ND4 11778G > A mutation, which affects structural components of complex I, is the most prevalent LHON-associated mitochondrial DNA (mtDNA) mutation worldwide. The m.11778G > A mutation is the primary contributor underlying the development of LHON and X-linked PRICKLE3 allele (c.157C > T, p.Arg53Trp) linked to biogenesis of ATPase interacts with m.11778G > A mutation to cause LHON. However, the lack of appropriate cell and animal models of LHON has been significant obstacles for deep elucidation of disease pathophysiology, specifically the tissue-specific effects. Using RGC-like cells differentiated from induced pluripotent stem cells (iPSCs) from members of one Chinese family (asymptomatic subjects carrying only m.11778G > A mutation or PRICKLE3 p.Arg53Trp mutation, symptomatic individuals bearing both m.11778G > A and PRICKLE3 p.Arg53Trp mutations and control lacking these mutations), we demonstrated the deleterious effects of mitochondrial dysfunctions on the morphology and functions of RGCs. Notably, iPSCs bearing only m.11778G > A or p.Arg53Trp mutation exhibited mild defects in differentiation to RGC-like cells. The RGC-like cells carrying only m.11778G > A or p.Arg53Trp mutation displayed mild defects in RGC morphology, including the area of soma and numbers of neurites, electrophysiological properties, ATP contents and apoptosis. Strikingly, those RGC-like cells derived from symptomatic individuals harboring both m.11778G > A and p.Arg53Trp mutations displayed greater defects in the development, morphology and functions than those in cells bearing single mutation. These findings provide new insights into pathophysiology of LHON arising from RGC deficiencies caused by synergy between m.11778G > A and PRICKLE3 p.Arg53Trp mutation.

## Graphical abstract

**Graphical Abstract:**
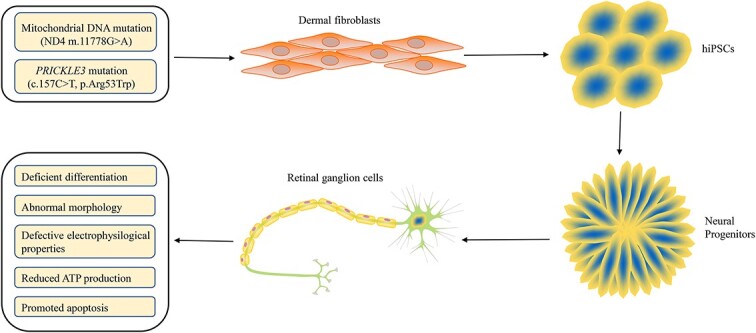

## Introduction

Leber’s hereditary optic neuropathy (LHON) is the most common example of maternal inheritance of eye diseases caused by mutations in mitochondrial DNA (mtDNA) (1–3). Clinically, it manifests as an acute or subacute, usually bilateral, severe central vision loss due to the degeneration of the retinal ganglion cells (RGC) layer and optic nerve axons, mainly affecting the small caliber fibers of the papillomacular bundle (2–5). The mtDNA mutations affecting structural components of NADH: ubiquinone oxidoreductase (complex I) accounted for the majority of LHON cases worldwide (6–12). The ND4 11778G > A mutation, which affects structural components of complex I, is the most prevalent LHON-associated mitochondrial DNA mutation worldwide (6–10). The matrilineal relatives carrying the m.11778G > A mutation exhibited a wide range of phenotypic heterogeneity including the severity, age-at-onset and penetrance of optic neuropathy (13–15). The primary defect in the LHON-linked mtDNA mutation was a failure in the activity of complex I, thereby causing the respiratory deficiency, diminishing ATP synthesis and an increasing generation of reactive oxygen species (16–21). The incomplete penetrance and gender bias in patients presenting with optic neuropathy suggests nuclear modifier genes such as X-linked modifier necessary for the phenotypic expression of LHON-associated mtDNA mutations (22,23). Our recent study demonstrated several LHON families manifested by synergic interaction between the m.11778G > A mutation and mutated modifiers *YARS2* encoding mitochondrial tyrosyl-tRNA synthetase and X-linked modifier *PRICKLE3* encoding a mitochondrial protein linked to biogenesis of ATPase (18,24,25). In particular, the *PRICKLE3* allele (c.157C > T, p. Arg53Trp) acted in synergy with m.11778G > A mutation and then deteriorated the mitochondrial dysfunctions necessary for the expression of LHON (25). However, the lack of appropriate cell and animal models of LHON has been significant obstacles for deep elucidation of disease pathophysiology, specifically the tissue-specific effects (26,27).

The use of human-induced pluripotent stem cells (hiPSCs) derived from patients to obtain terminally differentiated RGCs and neurons is a revolutionary approach to understanding pathogenic mechanisms of retinal disorders (28–34). To further elucidate the pathophysiology of LHON-linked m.11778G > A and PRICKLE3 p.Arg53Trp mutations, we generated the induced pluripotent stem cells (iPSCs) derived from matrilineal relatives of one Chinese family (asymptomatic carriers carrying only the m.11778G > A mutation or PRICKLE3 p.Arg53Trp mutation, symptomatic individuals bearing both m.11778G > A and heterozygous or hemizygous PRICKLE3 p.Arg53Trp mutations), and married-in control lacking these mutations (25,30,31). These iPSCs were differentiated into neural progenitor cells and subsequently RGC-like cells using a stepwise differentiation procedure (32,33). These RGC-like cells were assessed for effect of m.11778G > A and PRICKLE3 p.Arg53Trp mutations on the morphology and electrophysiological properties. The tissue-specific effects of m.11778G > A and PRICKLE3 p.Arg53Trp mutation on oxidative phosphorylation system (OXPHOS) was further evaluated for mitochondrial ATP contents using RGC-like cells, iPSCs and dermal fibroblasts derived from different genotypes. To evaluate if the m.11778G > A and PRICKLE3 p.Arg53Trp mutations affected the apoptotic processes, we examined the apoptotic state of mutant and control RGC-like cells by immunofluorescence.

## Results

### Generation of iPSCs derived from members of a Chinese family with LHON

**Figure 1:**
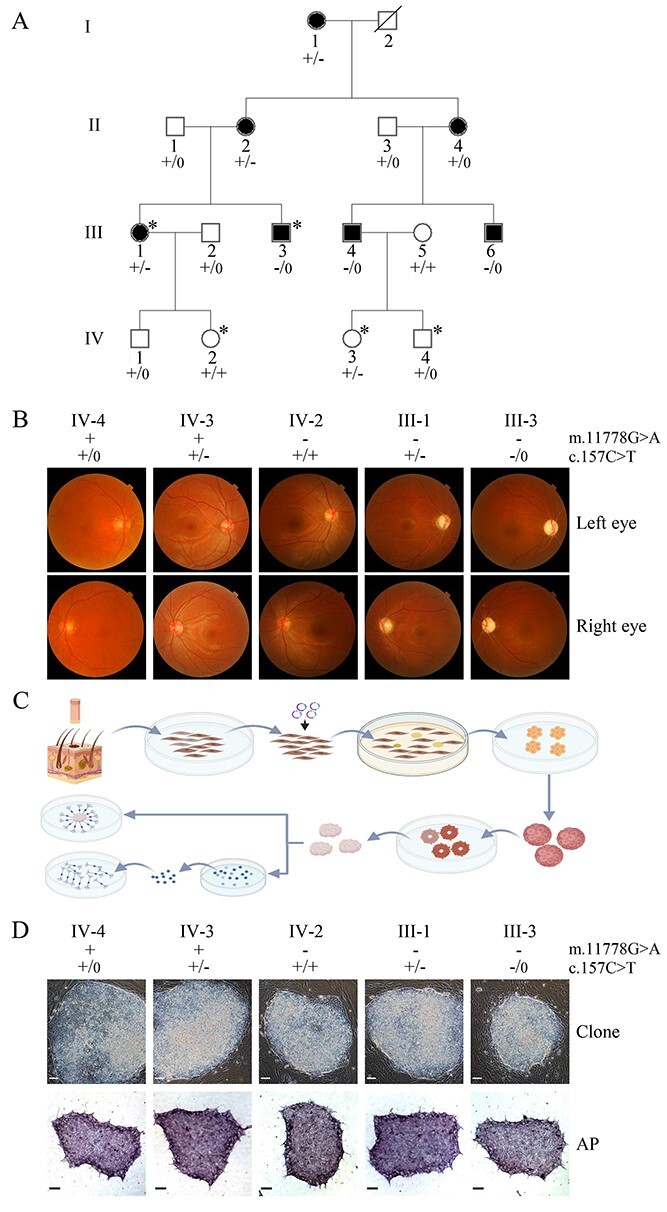
Generation of patient-derived iPSCs from human dermal fibroblasts. (**A**) Portion of one Chinese pedigree family (2. Individuals harboring hemizygous (−/0), heterozygous (+/−) PRICKLE3 (c.157C > T, p.Arg53Trp) mutation and wild-type (+/+ or +/0) were indicated. Individuals related to this study were marked with^*^. (**B**) Fundus photograph of five members in this family. Images were taken by the fundus photography (Cannon CR6-45NM fundus camera). (**C**) Schematic diagram illustrating that patient-derived iPSCs with various genotypes were generated from human dermal fibroblasts and then differentiated to neural progenitor cells or retinal ganglion cells. (**D**) Microscopy and alkaline phosphatase staining of iPSCs. The images above were the morphology of iPSCs clones on day 24 from the reprogramming start, surrounded by undifferentiated dermal fibroblasts, and the images below were the alkaline phosphatase staining of iPSCs clones cultured in a feeder-free condition. Scale bars = 100 μm.

To investigate the tissue-specific effects of m.11778G > A or PRICKLE3 p.Arg53Trp mutations on development and function of RGCs, we generated iPSCs from dermal fibroblasts donated by members of one Chinese family with LHON [vision-impaired subjects (III-1 and III-3 with both m.11778G > A with heterozygous or hemizygous PRICKLE3 p.Arg53Trp mutations), vision normal subjects (IV-2 bearing only m.11778G > A mutation, IV-3 harboring only PRICKLE3 p.Arg53Trp mutation and IV-4 lacking these mutations)] (Fig. 1A and B) (25). All dermal fibroblasts used for this investigation were analyzed for the presence and levels of the m.11778G > A mutation by both Sanger sequence and RFLP analyses. As shown in Supplementary Material, Figure S1, the m.11778G > A mutation was present in homoplasy in cell lines derived from subjects III-1, III-3 and IV-2 but absent in cell lines derived from the IV-3 and IV-4. These iPSCs were differentiated into neural progenitor cells and finally RGC-like cells using a stepwise differentiation procedure as shown for morphological and physiological characterizations (Fig. 1C). As a result, five iPSCs lines were generated from the fibroblasts of members of Chinese family by electroporation expression vectors bearing reprogramming factors. All iPSCs exhibited the morphological characteristics of human ESCs including the staining positive for alkaline phosphatase (Fig. 1D), and expression of pluripotent markers including NANOG, OCT4, TRA1-60, SOX2 and SSEA4 (Supplementary Material, Fig. S2A and B). Furthermore, the karyotyping analysis of all iPSCs at P10 or P20 revealed the absence of chromosomal abnormalities (Supplementary Material, Fig. S2C). Embryoid bodies (EBs) and teratomas were formed, and both of them differentiated into cells of all three germ layers (Supplementary Material, Fig. S2D, E). These results revealed that we successfully generated five iPSCs lines.

### Deficient differentiations of iPSCs into RGC-like cells

**Figure 2:**
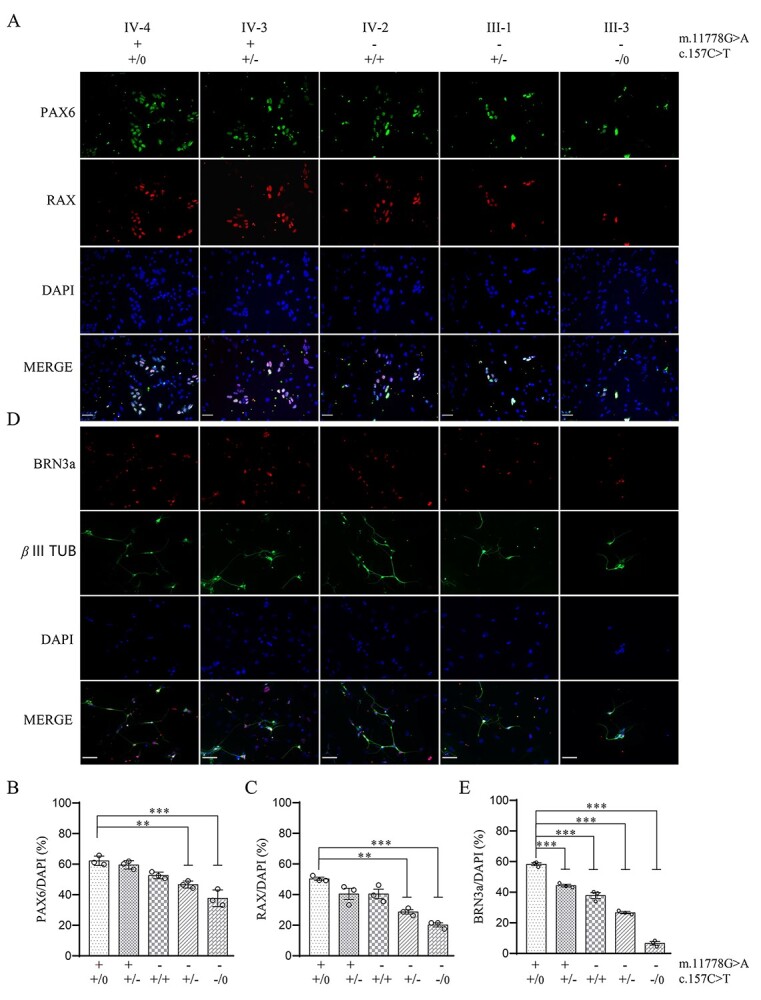
Deficient differentiation of iPSCs into RGC-like cells. (**A**) iPSCs from different genotypes were induced toward optic neuronal progenitors, and then staining with neural progenitor markers PAX6 (green) and RAX (red). Nuclei were stained with DAPI. Scale bars = 50 μm. (**B**) The proportion of PAX6 positive cells among the DAPI-positive cells on day 14, *n*≧3. (**C**) The proportion of RAX positive cells among the DAPI-positive cells on day 14, *n*≧3. (**D**) Generations of retinal ganglion were confirmed by immunostaining with retinal ganglion cells markers: BRN3a (red) and *β* III TUBULIN (green). Nuclei were stained with DAPI. Scale bars = 50 μm. (**E**) The proportion of BRN3a-positive cells among the DAPI-positive cells. n≧3. Data are presented as mean ± standard error of mean (SEM), *P* indicates the significance, ^*^P < 0.05, ^*^^*^P < 0.01, ^*^^*^^*^P < 0.001; ns, no statistically significant by one-way ANOVA followed by Bonferroni’s *post hoc* test.

The iPSCs from different genotypes underwent directed differentiated into RGC-like cells. To determine whether the cells had started differentiating toward early optic neuronal lineage, we assessed the dynamic expression of a panel of lineage markers expressing during optic/placodal development (32–36). For immunocytochemistry analyses, we used a combined expression of PAX6 plus RAX, SOX2 plus NESTIN and TJP1 plus RAX to track the differentiation of iPSCs with five different genotypes towards optic neuronal progenitor cells (ONPs) (32,35) (Supplementary Material, Fig. S3A). Immunocytochemistry analyses showed that differentiated cells from iPSCs expressed PAX6, RAX, SOX2, NESTIN and TJP1 at day 14 (Fig. 2A, Supplementary Material, Fig. S3A). To evaluate the effect of m.11778G > A and PRICKLE3 p.Arg53Trp mutations on differentiation efficiency of iPSCs into ONPs, we measured the PAX6 and RAX positive cells from different genotypes. As shown in Fig. 2B and C, the percentages of PAX6 positive-staining cells derived from subjects IV-4, IV-3, IV-2, III-1 and III-3 were 62.2, 59.5, 52.7, 46.7 and 37.7%, respectively, while those of RAX positive-staining cells from above genotypes were 50.3, 40.5, 40.4, 28.7 and 20.2%, respectively. These results implied that iPSCs bearing only m.11778G > A or PRICKLE3 p.Arg53Trp mutation exhibited milder defects in differentiation of neuronal progenitor cells than those in cells carrying both m.11778G > A and PRICKLE3 p.Arg53Trp mutations.

These optic progenitor cells were then induced RGC-like cell differentiation. After growing 28 days, differentiated cells with typical neuron morphology in all five groups were showed by immunocytochemical staining with retinal ganglion cell markers BRN3a and *β* III TUBULIN (Fig. 2D) (32,35). To assess the effect of m.11778G > A and PRICKLE3 p.Arg53Trp mutations on differentiation efficiencies of ONPs into RGC-like cells, we measured the BRN3a positive-staining cells from different genotypes. As shown in Figure 2E, the percentages of BRN3a positive-staining cells derived from subjects IV-4, IV-3, IV-2, III-1 and III-3 were 58.3, 44.3, 37.9, 26.6 and 6.7%, respectively. These indicated that only m.11778G > A or PRICKLE3 p.Arg53Trp mutation led to relatively mild defects in neuronal differentiation and PRICKLE3 p.Arg53Trp mutation aggravated the developmental defects of RGCs, caused by m.11778G > A mutation.

### Abnormal morphology of RGC-like cells

**Figure 3:**
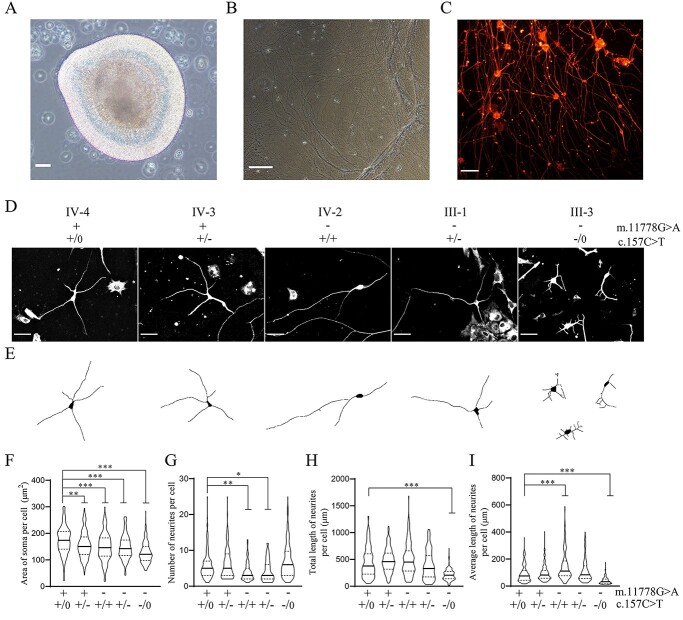
Abnormal morphology of RGC-like cells and axon elongation. (**A**) Classical morphology of early retinal organoid at day 21. Scale bars = 50 μm. (**B**) The axons and neural network formed by purified RGC-like cells in white phage. Scale bars = 100 μm. (**C**) The axons and neural network formed by purified RGC-like cells. Purified RGC-like cells were stained with *β* III TUBULIN (red). Scale bars = 100 μm. (**D**) Morphology of RGC-like cells. Cells were stained with *β* III TUBULIN on day 28. Scale bars = 20 μm. (**E**) Representative tracings of RGC-like cells by IMAGE J. (**F**) The soma size of RGC-like cells. *n*≧120. (**G**) The number of neurites per RGC-like cell. *n*≧120. (**H**) The total length of neurites per RGC-like cell. *n*≧120. (**I**) The average length of neurites per RGC-like cell. *n*≧120. Data are presented as mean ± standard error of mean (SEM), *P* indicates the significance, ^*^P < 0.05, ^*^^*^P < 0.01, ^*^^*^^*^P < 0.001; ns, no statistically significant by one-way ANOVA followed by Bonferroni’s *post hoc* test.

Typical RGC-like cells for morphology and function characterization were isolated from neurosphares exhibiting early retinal organoids morphology in day 21 (Fig. 3A) (29,37). After treating neurosphares from different genotypes with Accutase for 30 minutes, dispersed early retinal cells suspension were transferred to gelatin coated dishes for a 30 minutes pre-attachment, and the RGC-enriched supernatant were retransferred to new PDL/laminin-coated dishes for one week. All purified RGCs displayed the typical morphology, including extending long axons and forming neural network (Fig. 3B). These RGC-like cells were further verified by immunocytochemical staining with retinal ganglion cell markers BRN3a, *β* III TUBULIN, SNCG, THY1, ATOH7 and ISL1 (Fig. 3C, Supplementary Material, Fig. S3B). The effects of m.11778G > A and PRICKLE3 p.Arg53Trp mutations on morphological properties of RGC-like cells were assessed by immunochemical staining with *β* III TUBULIN in RGC-like cells. More than 120 differentiated cells with the most typical neuronal morphology in each group were analyzed by IMAGE J. As shown in Fig. 3D and E, the RGC-like cells bearing only m.11778G > A mutation exhibited more severe effect on morphology than those harboring only PRICKLE3 p.Arg53Trp mutation. Notably, RGC-like cells carrying both m.11778G > A and heterozygous or hemizygous PRICKLE3 p.Arg53Trp mutations displayed greater defects in soma and neurites than those cells bearing only single mutation. As shown in Figure 3F, the average areas of soma in the RGC-like cells carrying only PRICKLE3 p.Arg53Trp mutation, m.11778G > A mutation, both m.11778G > A and heterozygous or hemizygous PRICKLE3 p.Arg53Trp mutations and lacking these mutations were 155.8, 148, 148.2, 127.4 and 174.2 μm^2^, respectively. As shown in Figure 3G, the average numbers of neurites per RGC-like cells carrying only PRICKLE3 p.Arg53Trp mutation, m.11778G > A mutation, both m.11778G > A and heterozygous or hemizygous PRICKLE3 p.Arg53Trp mutations and lacking these mutations were 6.3, 4.1, 4.3, 7 and 5.9, respectively. As shown in Figure 3H, the total lengths of neurites per cell carrying only PRICKLE3 p.Arg53Trp mutation, m.11778G > A mutation, both m.11778G > A and heterozygous or hemizygous PRICKLE3 p.Arg53Trp mutations and lacking these mutations were 483.3, 503.6, 394.5, 215.6 and 449.4 μm, respectively. As shown in Figure 3I, the average lengths of neurites per cell carrying only PRICKLE3 p.Arg53Trp mutation, m.11778G > A mutation, both m.11778G > A and heterozygous or hemizygous PRICKLE3 p.Arg53Trp mutations and lacking these mutations were 96.7, 153.6, 113, 44.3 and 92.2 μm, respectively. These suggested that the PRICKLE3 p.Arg53Trp mutation aggravated the defects in morphological properties of the RGCs associated with m.11778G > A mutation.

### Defects in electrophysiological properties

**Figure 4:**
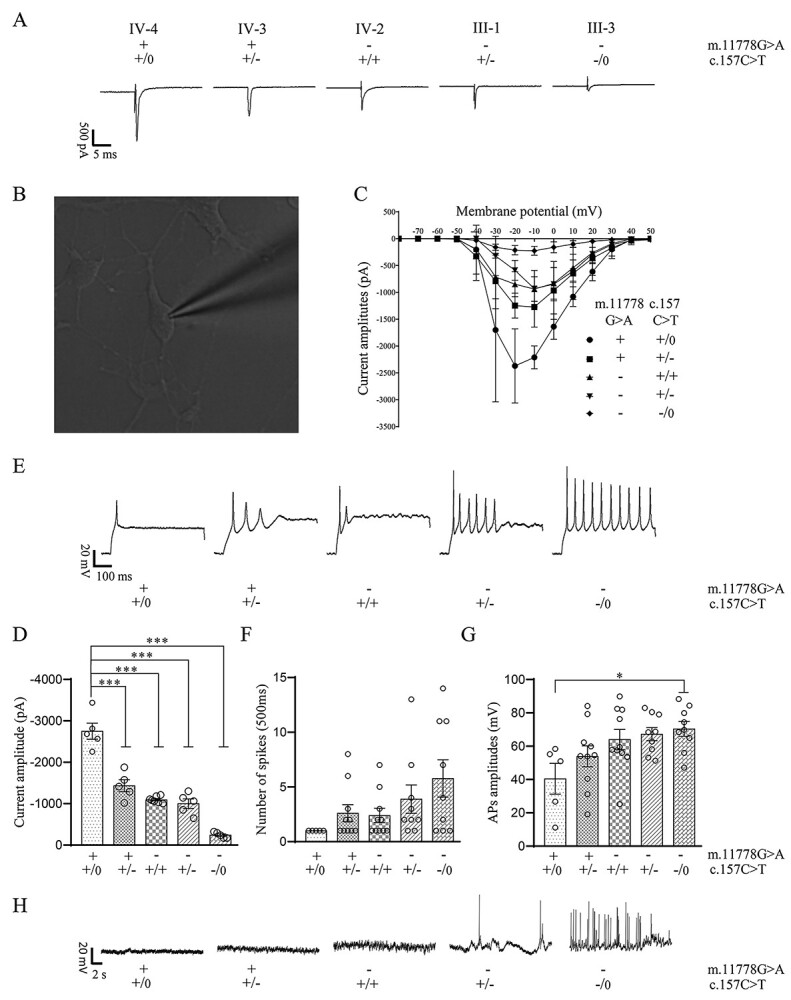
The electrophysiological properties of RGC-like cells. (**A**) Inward sodium ionic recording of solitary RGC-like cells in voltage-clamp mode. (**B**) RGC with recording microelectrode attached. (**C**) I-V curves displayed decreased inward current of RGC-like cells bearing mutations. *n*≧5. (**D**) Inward sodium current amplitudes of RGC-like cells. *n*≧5. (**E**) Patch-clamp recordings of evoke APs of RGC-like cells. (**F**) Evoke APs fired numbers of RGC-like cells. *n*≧5. (**G**) The amplitudes of evoke APs of RGC-like cells. *n*≧5. (**H**) The spontaneous APs fired by RGC-like cells. Data are presented as mean ± standard error of mean (SEM), *P* indicates the significance, ^*^P < 0.05, ^*^^*^P < 0.01, ^*^^*^^*^P < 0.001; ns, no statistically significant by one-way ANOVA followed by Bonferroni’s *post hoc* test.

To examine the electrophysiological properties, RGC-like cells with the typical neuronal morphology (cell body and neurites) in each genotype were first analyzed by whole-cell patch-clamp recordings (Fig. 4B) (32,38–40). Voltage-clamp recordings from RGC-like cells bearing only PRICKLE3 p.Arg53Trp mutation, m.11778G > A mutation, both m.11778G > A and heterozygous or hemizygous p.Arg53Trp mutations exhibited the various decreases in peak sodium conductance, as compared with those lacking these mutations (Fig. 4A and C). As shown in Figure 4D, the current amplitudes in the RGCs bearing only PRICKLE3 p.Arg53Trp mutation, m.11778G > A mutation, both m.11778G > A and heterozygous or hemizygous PRICKLE3 p.Arg53Trp mutations were 52.1, 39.9, 36.4 and 8.7% of those in control cells lacking these mutations, respectively.

These RGC-like cells from various genotypes were further characterized using electrophysiological analyses of the evoked action potentials (Aps) (Fig. 4E). RGC-like cells bearing only PRICKLE3 p.Arg53Trp mutation, m.11778G > A mutation, both m.11778G > A and heterozygous or hemizygous PRICKLE3 p.Arg53Trp mutations exhibited the wide range of increased numbers of AP firings (Fig. 4F) and AP amplitude (Fig. 4G), respectively. Subsequently, the ability of RGC-like cells to fire spontaneous APs was examined, with no spontaneous activity detected in RGC-like cells bearing only single mutation or lacking the mutations, but with spontaneous APs elicited in those carrying both m.11778G > A and heterozygous or hemizygous PRICKLE3 p.Arg53Trp mutations (Fig. 4H). These data demonstrated that the m.11778G > A or hemizygous PRICKLE3 p.Arg53Trp mutations impaired electrophysiological properties, which are the characteristics of functional neurons such as the RGC-like cells.

### Reduced ATP contents and promoting apoptosis

**Figure 5:**
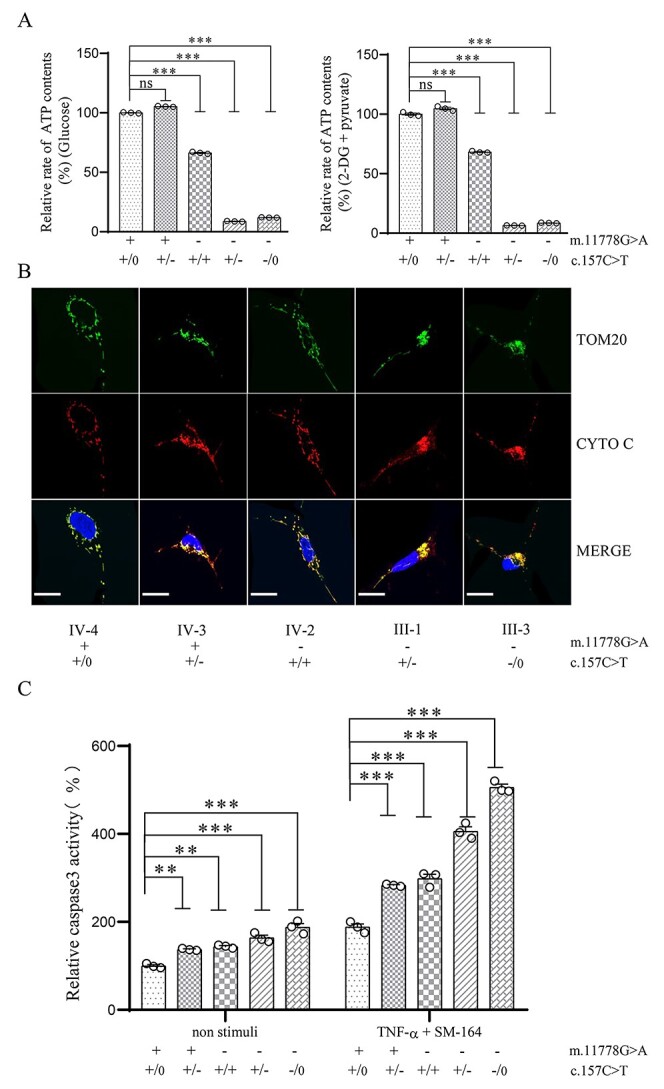
The analysis of ATP contents and apoptosis. (**A**) Measurement of cellular and mitochondrial ATP levels using bioluminescence assay. RGC-like cells were incubated with 10 mM glucose or 5 mM 2-deoxy-D-glucose plus 5 mM pyruvate to determine ATP generation under mitochondrial ATP synthesis. Average rates of ATP level per cell line in mitochondria are shown. (**B**) Analysis of apoptosis. The distributions of Cytochrome c from RGC-like cells were visualized by immunofluorescent labeling with TOM20 antibody conjugated to Alex Fluor 488 (green) and Cytochrome c antibody conjugated to Alex Fluor 594 (red) analyzed by confocal microscopy. DAPI-stained nuclei were identified by their blue fluorescence. Scale bars = 20 μm. (**C**) Measurement of Caspase 3 activity in the presence and absence of toxic TNF-α and SM-164 stimuli. TNF-α and SM-164 are effective toxic stimulus for apoptosis (46,47). RGC-like cells from different genotypes were treated with TNF-α + SM-164 using Apoptosis Inducer Kit (Beyotime) to induce apoptosis. Caspase-3 activity was measured using the Caspase-3 Activity Assay Kit (Beyotime). Three independent experiments were made for each cell line. Data are presented as mean ± standard error of mean (SEM), *P* indicates the significance, ^*^P < 0.05, ^*^^*^P < 0.01, ^*^^*^^*^P < 0.001; ns, no statistically significant by one-way ANOVA followed by Bonferroni’s *post hoc* test.

To test tissue-specific effects of PRICKL3 p.Arg53Trp and m.11778G > A mutations on oxidative phosphorylation, we used the luciferin/luciferase assay to examine the mitochondrial and total cellular ATP contents in mutant and control cells. Populations of cells were incubated in the media in the presence of glucose (reflects total cellular ATP contents), or 2-deoxy-D-glucose with pyruvate (reflects mitochondrial ATP contents) (41,42). As shown in Figure 5A, the levels of mitochondrial ATP contents in the RGC-like cells harboring only heterozygous PRICKLE3 p.Arg53Trp, m.11778G > A, both m.11778G > A and heterozygous or hemizygous PRICKLE3 p.Arg53Trp mutations were 73.3, 68.2, 6.5 and 8.6% of average values of control RGC-like cells lacking these mutations. By contrast, the mutant fibroblast cell lines exhibited mild decreases and mutant iPSCs revealed the various increases in the levels of mitochondrial ATP contents, respectively, as compared with control cells (Supplementary Material, Fig. S4). Furthermore, the levels of total cellular ATP contents in the RGC-like cells carrying only heterozygous PRICKLE3 p.Arg53Trp, m.11778G > A, both m.11778G > A and heterozygous or hemizygous PRICKLE3 p.Arg53Trp mutations were 73.5, 66.3, 8.7 and 11.9% of average values of control RGC-like cells, respectively (Fig. 5A). However, the levels of total cellular ATP contents in mutant fibroblast cell lines were comparable with those measured in the control cell lines, while mutant iPSCs displayed mild increases in the levels of total cellular ATP contents (Supplementary Material, Fig. S4).

Deficient activities of oxidative phosphorylation have been linked to protection against certain apoptotic stimuli (43,44). To evaluate if the m.11778G > A and PRICKLE3 p.Arg53Trp mutations affected the apoptotic processes, we examined the apoptotic state of mutant and control RGC-like cells by immunofluorescence (45,46). As shown in Figure 5B, the immunofluorescence patterns of double-labeled cells with rabbit monoclonal antibody specific for the Cytochrome c and mouse monoclonal antibody to TOM20 revealed mildly increased levels of Cytochrome c in the mutant cells bearing only m.11778G > A or PRICKLE3 p.Arg53Trp mutations, and markedly increases of Cytochrome c in the RGC-like cells bearing both m.11778G > A and heterozygous or hemizygous PRICKLE3 p.Arg53Trp mutations, as compared with control RGC-like cells. We further evaluated the effect of m.11778G > A mutation and PRICLKE3 mutation on apoptosis by measurement of Caspase 3 activity in the presence and absence of toxic TNF-α and SM-164 stimuli (47–49). As shown in Figure 5C, relative activity of Caspase 3 in the RGC-like cells harboring only heterozygous PRICKLE3 p.Arg53Trp, m.11778G > A, both m.11778G > A and heterozygous or hemizygous PRICKLE3 p.Arg53Trp mutations were 137.2, 144, 163.6 and 187.8% of average values of control RGC-like cells lacking these mutations. Notably, the mutant RGC-like cells were more sensitive to toxic stimuli than those in control RGC-like cells. After 2 hours treatment with apoptotic stimuli TNF-α and SM-164, the levels of Caspase 3 activity in control RGC-like cells and mutant RGC-like cells carrying only heterozygous PRICKLE3 p.Arg53Trp, m.11778G > A, both m.11778G > A and heterozygous or hemizygous PRICKLE3 p.Arg53Trp mutations were 188.1, 283.3, 298.5, 405.9 and 505.8% of average values of control RGC-like cells in the absence of toxic stimuli, respectively (Fig. 5C). These are strong evidences that PRICKLE3 p.Arg53Trp or m.11778G > A itself promoted apoptosis and the PRICKLE3 p.Arg53Trp mutation worsened the defects in apoptosis in the RGC-like cells associated with m.11778G > A mutation.

## Discussion

The tissue specificity of LHON-linked mtDNA mutations remains largely elusive. The pathogenic mechanism behind the tissue-specific manifestations are likely to involve RGC-specific mitochondrial functions. RGCs are highly polarized neurons that produce axons extending to 5 cm in length from the cell soma (50). RGC-like cells differentiated from those iPSCs-derived LHON patients provided excellent cell models to understand tissue specificity of LHON-linked mtDNA mutations. In fact, the LHON-linked m.11778G > A mutation conferred only relatively mild defects in mitochondrial complex I activity and ATP contents but not sufficient to produce the LHON phenotype (17,18), while an LHON-nuclear modifier PRICKLE3 p.Arg53Trp mutation resulted in the defective assembly, stability and function of ATP synthase but was itself insufficient to produce an LHON phenotype (25). The PRICKLE3 p.Arg53Trp mutation acted in synergy with m.11778G > A mutation and deteriorated mitochondrial dysfunctions necessary for the expression of LHON (25). Using the RGC-like cells differentiated from those iPSCs from members of one Chinese family (asymptomatic carriers carrying only the m.11778G > A mutation or PRICKLE3 p.Arg53Trp mutation, symptomatic individuals bearing both m.11778G > A and heterozygous or hemizygous PRICKLE3 p.Arg53Trp mutations and married-in control lacking these mutations), we investigated the effects of m.11778G > A, PRICKLE3 p.Arg53Trps mutation and their synergy on the morphology and functions of RGCs. In this study, we showed that those iPSCs derived from asymptomatic individuals harboring only m.11778G > A mutation or PRICKLE3 p.Arg53Trps exhibited mild defects in the development, morphology and functions of RGCs. Strikingly, those iPSCs derived from symptomatic individuals bearing both m.11778G > A and heterozygous or hemizygous PRICKLE3 p.Arg53Trp mutations) displayed severe defects in the development, morphology and functions of RGCs.

The RGCs are particularly vulnerable to neurodegeneration related to mitochondrial dysfunction (50,51). In this study, we demonstrated that mitochondrial dysfunction altered the iPSCs differentiation to RGCs. The m.11778G > A or PRICKLE3 p.Arg53Trp mutation exhibited mild defects in neuronal differentiation, evidenced by approximately 20% decreases in the RAX positive-staining cells in ONPs carrying only m.11778G > A or PRICKLE3 p.Arg53Trp mutation and 25–35% reductions in BRN3a positive-staining cells in mutant RGC-like cells bearing only m.11778G > A or p.Arg53Trp mutation, as compared with those in control ONPs and RGC-like cells. Strikingly, there were 51% decreases in the RAX positive-staining cells in ONPs carrying both m.11778G > A and p.Arg53Trp mutations and 72% reductions in BRN3a positive-staining cells in mutant RGC-like cells bearing both m.11778G > A and p.Arg53Trp mutations, as compared with those in control ONPs and RGC-like cells. These highlighted that the p.Arg53Trp mutation acted in synergy with m.11778G > A mutation and deteriorated the developmental defects of RGCs necessary for the expression of LHON.

The mitochondrial dysfunctions affected the neuronal morphology (51,52). In particular, the defective neurite outgrowth was observed in the RGC-like cells derived from patients carrying the m.11778G > A mutation (33). In this study, the m.11778G > A mutation affected the RGC morphology, evidenced by 15% decrease in the area of soma and 30% reductions in the numbers of neurites in mutant RGC-like cells bearing the m.11778G > A mutation. The RGC-like cells carrying the p.Arg53Trp mutation displayed 11% reduction in the area of soma but no differences in the numbers of neurirtes, as compared with those in the control RGC-like cells lacking these mutations. However, the RGC-like cells harboring both m.11778G > A and p.Arg53Trp mutations exhibited greater defects in RGC morphology including the area of soma and the numbers of neurirtes than those in the cells carrying only m.11778G > A or p.Arg53Trp mutation.

The ability of neurons to properly initiate APs is fundamental for nervous system function and information processing. In this study, we demonstrated that these mutant RGC-like cells exhibited defects in electrophysiological properties, including decreasing current amplitudes, increasing numbers of spikes and raised AP amplitudes. The RGC-like cells bearing the m.11778G > A mutation revealed 40% decrease in the current amplitudes, 140% increases in the numbers of AP spikes and raising 58% AP amplitude, while the RGC-like cells carrying the p.Arg53Trp displayed 52% reduction in the current amplitudes, 160% increases in the AP spikes and raising 33% AP amplitude. Notably, RGC-like cells bearing both m.11778G > A and p.Arg53Trp mutation exhibited greater defects in electrophysiological properties, including decreasing current amplitudes, increasing numbers of spikes and raised AP amplitudes than those harboring only m.11778G > A or p.Arg53Trp mutation. Furthermore, RGC-like cells bearing both m.11778G > A and p.Arg53Trp mutation displayed spontaneous AP, in contrast with no spontaneous activity in RGC-like cells bearing only single mutation. These data demonstrated that the synergy of PRICKLE3 p.Arg53Trp mutation with m.11778G > A mutation led to the defects in the morphology and function of RGCs necessary for the expression of LHON.

The iPSCs-derived RGCs from patients carrying m.11778G > A mutation displayed defective mitochondrial biogenesis, decreased basal respiration, increased oxidative stress and increased apoptosis (28,29,33). Here, the RGC-like cells bearing only m.11778G > A or p.Arg53Trp mutation exhibited mild decreases in the mitochondrial ATP contents, comparable with those in fibroblast, lymphoblastoid or cybrid cell lines (17,20,25). Strikingly, the RGC-like cells harboring both m.11778G > A and heterozygous or hemizygous PRICKLE3 p.Arg53Trp mutations revealed approximately 90% decreases in the mitochondrial ATP contents, in contrast with 40–50% decreases observed in these fibroblast or lymphoblastoid cell lines carrying both m.11778G > A and p.Arg53Trp mutations (25). Deficient activities of oxidative phosphorylation promoted apoptotic process (43,44). We showed that m.11778G > A or PRICKLE3 p.Arg53Trp mutation mildly affected the apoptosis, evidenced by mild increasing levels of Cytochrome c and Caspase 3 activity in the RGC-like cells bearing only m.11778G > A or PRICKLE3 p.Arg53Trp mutations. However, the RGC-like cells carrying both m.11778G > A and p.Arg53Trp mutations exhibited markedly increased apoptosis, supported by pronounced increases of Caspase 3 activity and Cytochrome c in the RGC-like cells. These mitochondrial dysfunctions caused by the combination of m.11778G > A with PRICKLE3 p.Arg53Trp mutations promoted apoptotic processes. These data demonstrated the tissue-specific effect of mitochondrial dysfunctions on the RGC deficiencies for the expression of LHON.

In summary, we demonstrated the impact of m.11778G > A, PRICKLE3 p.Arg53Trps mutation and their synergy on the morphology and functions of RGCs. The m.11778G > A mutation or PRICKLE3 p.Arg53Trps exhibited mild defects in the development, morphology and functions of RGCs but insufficient to produce LHON phenotype. The synergy of PRICKLE3 p.Arg53Trp mutation with m.11778G > A mutation led to severe defects in the development, morphology and functions of RGCs necessary for the expression of LHON. These findings provide new insights into pathophysiology of LHON that was manifested by RGC deficiencies arising for the mitochondrial dysfunctions due to the synergy between m.11778G > A mutation and nuclear modifier PRICKLE3 p.Arg53Trp mutation.

## Materials and Methods

### Generation of patient-derived iPSCs

Skin biopsies were donated from members of one Chinese pedigree [vision-impaired subjects (III-1 and III-3 with both m.11778G > A with heterozygous or hemizygous PRICKLE3 p.Arg53Trp mutations), vision normal subjects (IV-2 bearing only m.11778G > A mutation, IV-3 harboring only PRICKLE3 p.Arg53Trp mutation and IV-4 lacking these mutations)] (25). Human dermal fibroblasts were isolated from skin biopsies by IV collagenase (Roche) treatment using standard biosafety operations and cultured in DMEM-HG (Hyclone) supplemented with 10% FBS (Gibco), 1 X Glutamax (Gibco), 1X NEAA (Gibco), 50 μg/ml uridine (Sigma) and 1 mM/ml sodium pyruvate (Sigma). Patient-derived iPSCs were generated using modified protocol as previously described (30). Five hundred thousand fibroblasts at passage three were transfected 0.5 mg of each episomal plasmid (pCXLE-EGFP, pCXLE-hSK, pCXLE-hUL, pCXLE-hOCT3/4-shp53-F, pCXWB-EBNA1, Addgene) using Human Dermal Fibroblast Nucleofector™ Kit (LONZA) on a Nucleofector 2b device. This was defined as day 0. On day 7, fifty thousand transfected cells were seed into a well of 6-well plate coated with Matrigel (Corning) and grown on mTesR1 medium (Stemcell). During D1 to D11, 0.5 mM sodium butyrate (Sigma) was added to the culture medium. Well-formed colonies were manually picked and transferred into new matrigel-coated (Corning) dished and cultured in mTesR1 medium on day 24. When iPSCs were grown to a suitable confluence, Accutase (Stemcell) was used for iPSC passage.

### Differentiation of iPSCs to induced RGCs

iPSCs were stepwise differentiated to neural progenitor cells and retinal ganglion cells using previously described protocol with several modifications (32,33). All differentiations were performed before passage 15 of iPSCs. Briefly, iPSCs at 80% confluence were enzymatically dissociated into single cells by Accutase (Stemcell), two billion cells were cultured in suspension with mTesR1 medium (Stemcell) supplemented with 10% FBS (Gibco) to start the formation of embryoid bodies. This day was defined as day 0. The medium for embryoid bodies was gradually changed to N2 media containing a 1:1 mix of DMEM/F12 and Neurobasal supplemented with 1% N2 (Gibco), 1X Glutamax (Gibco), 1X NEAA (Gibco), 50 μg/ml uridine (Sigma), 1 mM/ml sodium pyruvate (Sigma), 1 ng/ml noggin (MCE) by day 1 and day 2. The medium was fully changed every day and 10 μM/ml Y27632 (MCE) was added into the medium from day 0 to day 7 to maintain cell survival. On day 7, embryoid bodies were planted onto 6-well plates coated with 0.1% gelatin (Sigma) and neural rosettes appeared after several days. On day 7 to day 14, a modified N2 media with 5 ng/ml noggin was used for differentiation and changed daily. Cells were used for analysis as neural progenitor cells on day 14. For further differentiation, neural rosettes clusters were selected detachment from adherent cell aggregates by STEMdiff™ Neural Rosette Selection Reagent treatment and cultured in suspension in B27 media containing a 1:1 mix of DMEM/F12 and Neurobasal supplemented with 2% B27 (Gibco), 1X NEAA (Gibco), 50 μg/ml uridine (Sigma), 1 mM/ml sodium pyruvate (Sigma), 5 ng/ml noggin (MCE), 10 μM/ml DAPT (MCE) and Fresh B27 media was changed every other day to allow the formation of neurospheres. Neurospheres were processed in different ways on day 21 to adapt to different analysis. In some cases, neurospheres were planted onto PDL/laminin-coated plates without additional treatment, and in other cases, neurospheres were collected and treated with Accutase (Stem cell) for 30 minutes to disperse the OV-like structure. The single cell populations were planted onto a gelatin-coated plate for 30 minutes. After pre-attachment, the RGC-enriched supernatant was transferred to PDL/laminin-coated plates. In all cases, fresh B27 media was half changed daily after attachment. Cells on day 28 were analyzed as matured RGC-like cells.

### Immunofluorescence assay

At the appropriate time point, various cells grown in coverslips were fixed with 4% paraformaldehyde for 20 minutes at room temperature. After three washes with phosphate-buffered saline (PBS), cells were permeabilized with 0.25% Triton X-100 for 10 minutes (53). After three washes with PBS, cells were incubated with 5% bovine serum albumin (BSA) blocking buffer for 1 hour at room temperature, followed by staining in primary antibody diluted in blocking buffer for 2 hours at room temperature. Subsequently, cells were washed with PBS four-time and then incubated with secondary antibodies (Alexa Fluor 488 or Alexa Fluor 594, abcom) diluted in blocking buffer for 1 hour and counterstained with DAPI solution for another 10 minutes at room temperature. Coverslips were mounted in anti-fluorescence quenching mounting medium and then microphotographed using an FV1000 confocal laser-scanning microscope (Olympus).

### Morphology of induced RGCs

For morphology analysis, dispersed and purified RGC-like cells were transferred on PDL/laminin-coated slides at a low density and matured RGC-like cells were fixed and stained with *β* III TUBULIN. More than one hundred random fields were photographed with a fluorescence microscope (Leica), details of neurites of cells displayed *β* III TUBULIN positive and without contact to other cells were recorded and analyzed by IMAGE J (54–56).

### Electrophysiological recording

RGC-like cells were transferred to PDL/laminin-coated slides at a low density. Solitary-induced RGCs were chosen to recording electrophysiological. A whole-cell patch-clamp technique was applied using a HEKA EP10 amplifier as described previously (40,57) and data were collected by Patchmaster software. Extracellular solution contained 140 mM NaCl, 1 mM MgCl_2_, 5 mM KCl, 2 mM CaCl_2_, 10 mM, HEPES and 10 mM glucose (pH 7.3). For voltage-clamp recording, internal solution contained 130 mM CsF, 10 mM NaCl, 10 mM HEPES, 1 mM and CsEGTA (EGTA in CsOH). Inward sodium current was recorded in voltage-clamp mode with a start holding potential of −80 mV and incremental steps of 10 up to 50 mV. For current-clamp recording, internal solution contained 140 mM KCl, 5 mM MgCl_2_, 2.5 mM CaCl_2_, 5 mM EGTA and 10 mM HEPES (pH 7.3). After establishing the whole-cell configuration, spontaneous APs activity was recorded immediately. For evoked APs, a 500 ms pulse duration after initiation of either step current injections with incremental steps of 2 pA up to 40 pA was recorded. Clampfit software was used for data analysis.

### Measurements of ATP contents

An enhanced ATP Assay Kit (Beyotime) was used for measurements for ATP levels according to the instructions from the manufacturer. Briefly, before analysis, cells for cellular ATP content measurement were cultured in solution contained 156 mM NaCl, 3 mM MgSO_4_, 3 mM KCl, 2 mM KH_2_PO_4,_ 2 mM CaCl_2,_ 20 mM HEPES (pH 7.4) at 37°C, while cells for mitochondrial ATP content measurement were cultured in solution contained 156 mM NaCl, 3 mM MgSO_4_, 3 mM KCl, 2 mM KH_2_PO_4,_ 2 mM CaCl_2,_ 20 mM HEPES (pH 7.4), 10 mM 2-DG and 5 mM sodium pyruvate for 2 hours at 37°C. Supernatants from lysed cells were taken for luciferase detected. The total cell mass or total protein mass were used to normalized.

### Assay for Cytochrome c release of RGC-like cells

RGC-like cells were fixed with 4% paraformaldehyde, permeabilized with 0.25% Triton X-100, blocked with 5% BSA, stained with TOM20 and Cytochrome c primary antibody and then stained with fluorescence secondary antibodies, counterstained with DAPI solution in sequence. Solitary-induced RGCs were microphotographed using FV1000 confocol laser-scanning microscope (Olympus) then the Mander’s Colocalization Coefficients (MCC) of CYTO C and TOM 20 were measured using Coloc 2 plugin in IMAGE J software (48,49).

### Caspase 3 activity assays

TNF-α and SM-164 are effective toxic stimulus for apoptosis (46,47). Cells were treated with (TNF-α + SM-164) according to the specifications of the Apoptosis Inducer Kit (Beyotime) to induce apoptosis. Caspase-3 activity was measured according to the specifications of the Caspase-3 Activity Assay Kit (Beyotime). The assay was based on the principle that Ac-DEVD-pNA (acetyl-Asp-Glu-Val-Asp p-nitroanilide) can be catalyzed by Caspase-3 and then produces pNA (p-nitroaniline), which gives a yellow color. Briefly, the detection samples were acquired by cell lysis and centrifugation at 4°C, then mixed and incubated with 0.2 mM Ac-DEVD-pNA at 37°C for 2 h. The absorbance of each sample at 405 nm was read and the Caspase-3 activity was calculated in combination with the standard curve and protein concentration measured by the Bradford assay.

### Statistics

Statistical analysis was performed using SPSS for statistical analysis to compare outcomes. For multiple comparisons, one-way ANOVA followed by Bonferroni’s post hoc test was performed. *P-*values of less than 0.05 were considered statistically significant.

### Study approval

Informed consent in writing prior to their participation in this study were obtained from members of families and control subjects, under protocols approved by the Ethic Committees of Zhejiang University School of Medicine. Furthermore, all experiments involving mice were approved by the Institutional Animal Care and Use Committee, Zhejiang University School of Medicine.

## Supplementary Material

supplement_data_clean_ddac190

## Data Availability

Representative experiments are shown in the Figures and supplemental materials. For any additional information, please contact the corresponding author.
