# Supplementary material for: Abnormal morphology and function in retinal ganglion cells derived from patients-specific iPSCs generated from individuals with Leber’s hereditary optic neuropathy
Source: Hum Mol Genet. 2022 Aug 10;32(2):231–43. doi: 10.1093/hmg/ddac190 (PMC9840204; doi:10.1093/hmg/ddac190)
Supplement: supplement_data_clean_ddac190 [file supplement_data_clean_ddac190.pdf]

Supplementary data for

Abnormal morphology and function in retinal ganglion cells derived from patients-specific iPSCs generated from individuals with Leber's hereditary optic neuropathy

Zhipeng Nie[1,2], Chenhui Wang[2], Jiarong Chen[1,2], Yanchun Ji[1,2], Hongxing Zhang[3], Fuxin Zhao[4], Xiangtian Zhou[4], and Min-Xin Guan[1,2,3,5,6]*

The supplemental data included the following information:

1. Supplemental Figure S1, S2, S3 and S4;
2. Supplemental Table S1, S2, and S3

Supplemental Method

Analysis of mitochondrial DNA

Genomic DNA was isolated from whole blood of participants using QIAamp DNA Blood Mini Kit (Qiagen, No.51104). The subject's DNA fragments spanning the ND4 genes were PCR amplified by use of oligodeoxynucleotides corresponding to mtDNA at positions 11,654-11,865 (1,2). Fragment was purified and then analyzed by direct sequencing. These sequence results were compared with the updated consensus Cambridge sequence (GenBank accession number: NC_012920) (3). These sequence results were compared with the updated consensus Cambridge sequence, as described above. To quantify the m.11778G>A mutation, the first PCR segments (803 bp) were amplified using genomic DNA as the template and oligodeoxynucleotides corresponding to mtDNA at positions 11,295-12,098, to rule out the coamplification of possible nuclear pseudogenes. Then, the second PCR product (212 bp) was amplified, using the first PCR fragment as the template and oligodeoxynucleotides corresponding to mtDNA at positions 11,654-11,865, and subsequently digested with the restriction enzyme *Tsp45*I as the G1178A mutation creates the site for this restriction enzyme (1). Equal amounts of various digested samples were then analyzed by electrophoresis through 7% polyacrylamide gel. The proportions of digested and undigested PCR product were determined by laser densitometry after ethidium bromide staining to determine if the m.11778G>A mutation is in homoplasmy in these subjects.

1. Qu, J., Li, R., Tong, Y., Hu, Y., Zhou, X., Qian, Y., Lu, F., and Guan MX. (2005) Only male matrilineal relatives with Leber's hereditary optic neuropathy in a large Chinese family carrying the mitochondrial DNA G11778A mutation. *Biochem. Biophys. Res. Commun.* , **328**, 1139-1145.
2. Jiang, P., Liang, M., Zhang, J., Gao, Y., He, Z., Yu, H., Zhao, F., Ji, Y., Liu, X., Zhang, M., Fu, Q., Tong, Y., Sun, Y., Zhou, X., Huang, T., *et al.* (2015) Prevalence of mitochondrial ND4 mutations in 1281 Han Chinese subjects with Leber's hereditary optic neuropathy. *Invest. Ophthalmol. Vis. Sci.*, **56**, 4778-4788.
3. Andrews, R.M., Kubacka, I., Chinnery, P.F., Lightowlers, R.N., Turnbull, D.M. and Howell, N. (1999) Reanalysis and revision of the Cambridge reference sequence for human mitochondrial DNA. *Nat. Genet.*, **23**, 147.

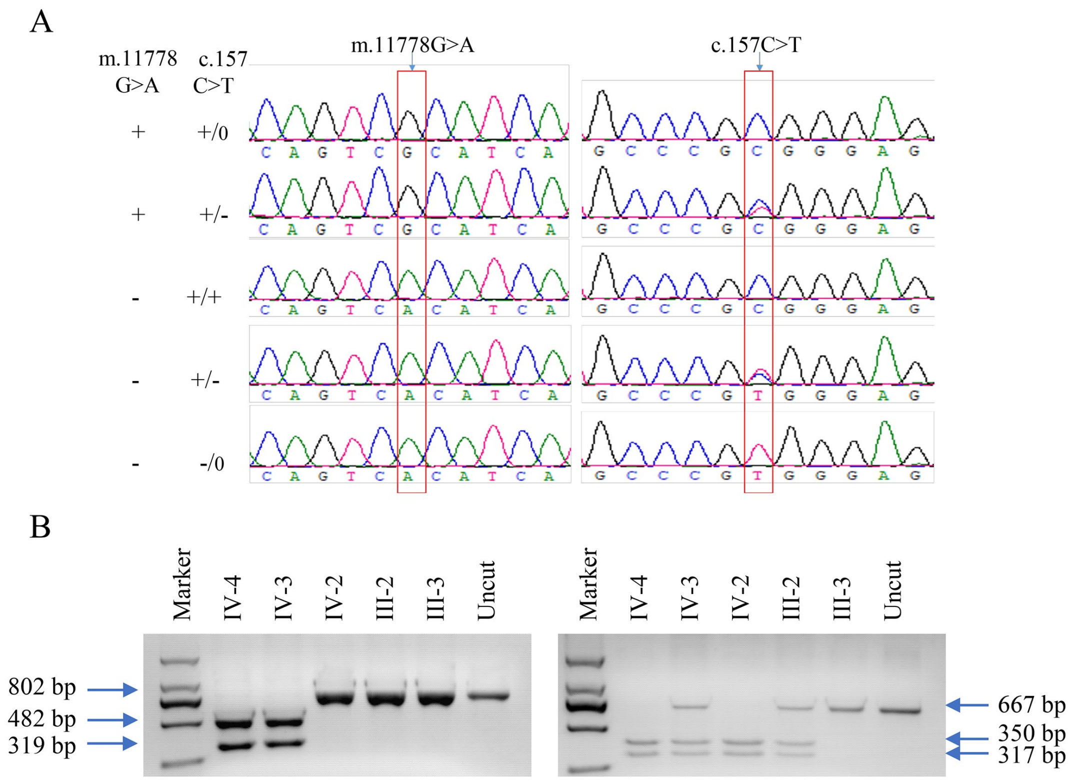

Supplemental Figure S1. Genotyping analysis. (A) Partial sequence eletropherograms of ND4 gene carrying the m.11778G>A mutation (left) and *PRICKLE3* harboring c.157C>T mutation from dermal fibroblasts driven from pedigree numbers harboring different gene mutations and wild-type. The arrow on left indicated the location of the nucleotide change at mitochondrial genome 11778, and the arrow on right indicated the location of the nucleotide change at *PRICKLE3* c.157. (B) RFLP analysis for the m.11778G>A mutation (left) and *PRICKLE3 c.157C>T* mutation (right) in dermal fibroblasts. Genotyping for the m.11778G>A mutation and *PRICKLE3 c.157C>T* mutation were PCR amplified and followed by digestion with the restriction enzyme *SfaN* I (for m.11778) or *Sac* II (for *PRICKLE3 c.157C>T*) and analyzed by electrophoresis in a 10% polyacrylamide gel stained with ethidium bromide

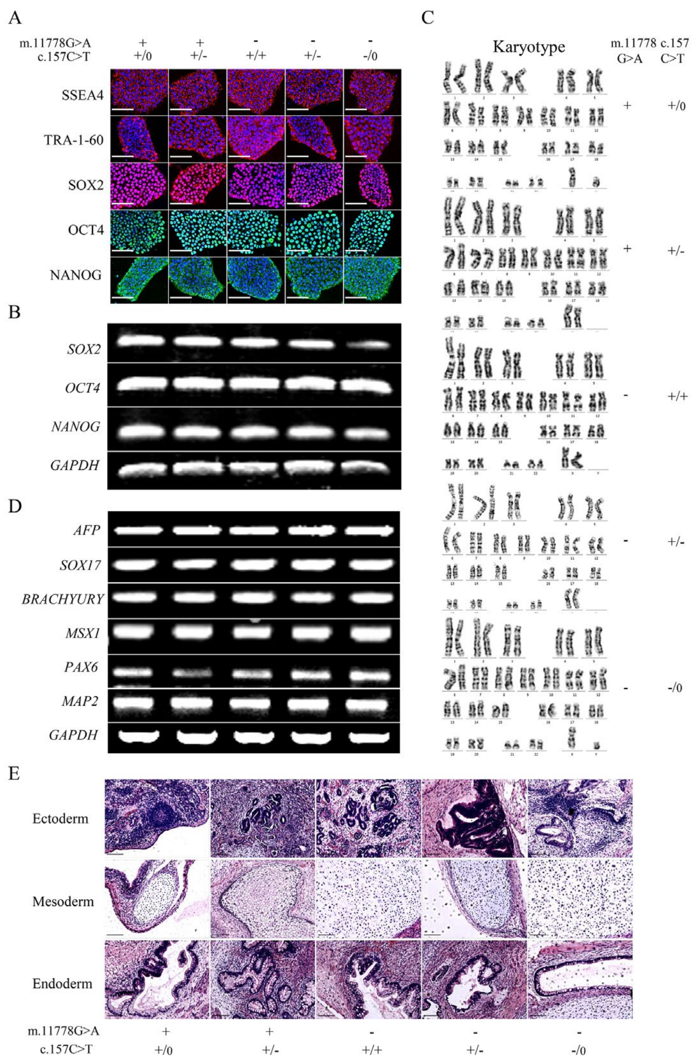

Supplement Figure S2: Characterization of iPSCs from human dermal fibroblasts. **(A)** Characterization of generated iPSCs in terms of pluripotency markers SSEA4, TRA-1-60, SOX2, OCT4, NANOG. Nuclei were stained with DAPI. Scale bars=100 µm. **(B)** RT-PCR analysis revealed the expression of pluripotency marker genes *SOX2, OCT4,* and *NANOG* in all iPSCs. **(C)** The karyotypes of iPSCs and all iPSCs displayed normal karyotypes at passage 20. **(D)** RT-PCR analysis of marker genes in different germ layers in iPSC-derived embryoid bodies. **(E)** Hematoxylin and eosin staining of iPSC-derived teratomas and the characteristic primitive tissue structure of three germ layers were detected: ectoderm (neuron rosettes), mesoderm (bone), endoderm (celenteron). Scale bars=100 µm.

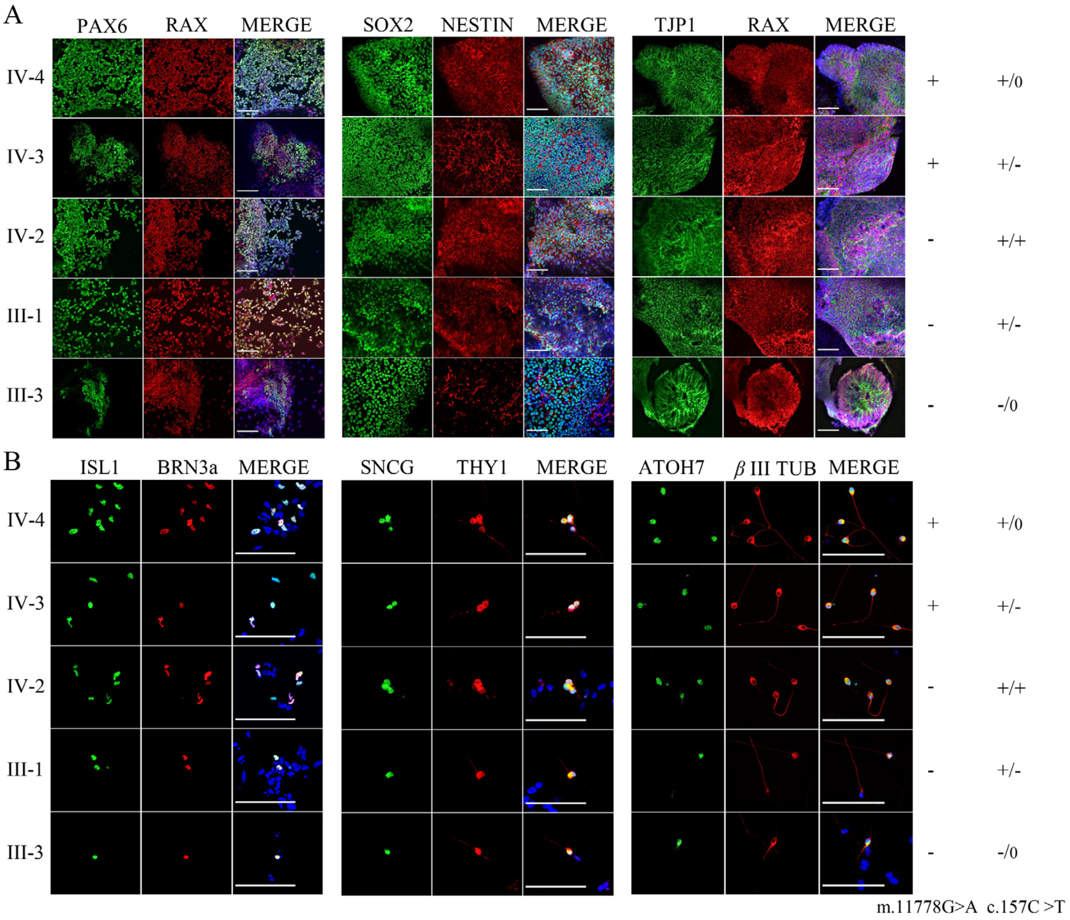

Supplement Figure S3: Characterization of neural progenitor cells and RGC-like cells. **(A)** Neural progenitor cells staining by neural progenitor cell markers PAX6, RAX, SOX2, NESTIN, TJP1. Nuclei were stained with DAPI. Scale bar=20 μm. **(B)** RGC-like cells were stained with retinal ganglion cell markers ISL1, BRN3a, SNCG, THY1, ATOH7, and β III TUBULIN. Nuclei were stained with DAPI. Scale bars=20 μm.

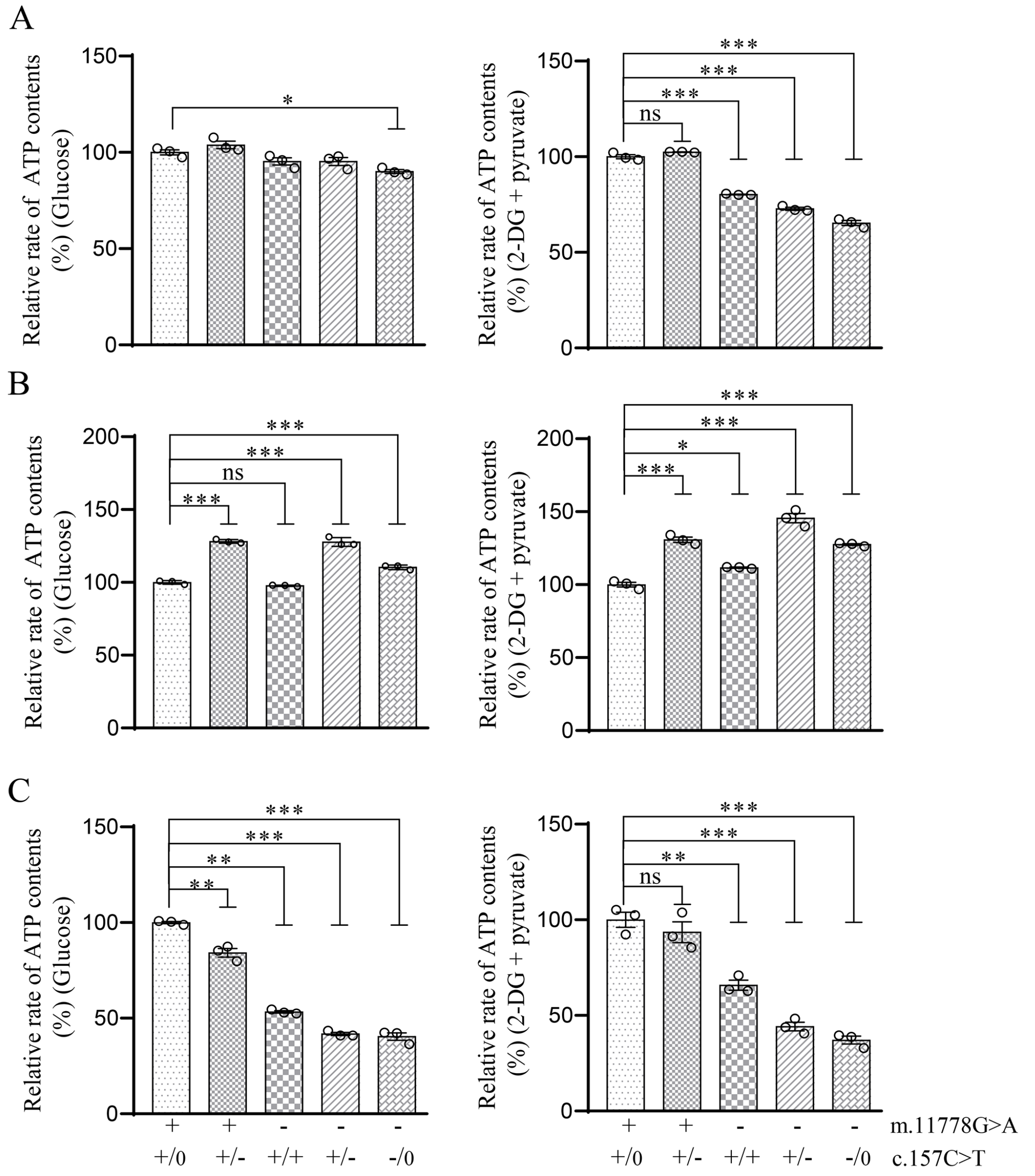

Supplement Figure S4: The energy metabolism dysfunction of fibroblasts and iPSCs. **(A)** Relative cellular ATP (left) and mitochondrial ATP (right) contents of fibroblasts. **(B)** Relative cellular ATP (left) and mitochondrial ATP (right) contents of iPSCs. **(C)** Relative cellular ATP (left) and mitochondrial ATP (right) contents of neural progenitor cells. Data are presented as mean± standard error of mean (SEM), *P* indicates the significance, **P*<0.05, ***P*<0.01, ****P*<0.001; ns, no statistically significant by one-way ANOVA followed by Bonferroni's post hoc test.

Supplement table S1: The genotypes and phenotypes of individuals related to this study.

Gender	m.11778 G>A	c.157C>T	Patient
male	+	+/0	NO
female	+	+/-	NO
female	-	+/+	NO
female	-	+/-	YES
male	-	-/0	YES

Supplement table S2: List of primers used in present study.

Primer	Forward sequence (5'-3')	Reverse sequence (5'-3')
PRICKLE3 c.157C>T	TAATAGGATTGGGGTGAACAGGC	TGAGGTCAATACATGCAGGAACC
m.11778 G>A	TCACTCTCACTGCCCAAGAA	GGAGAATGGGGGATAGGTGT
hrt *PRICKLE3*	GGGCCATCTGTGAGGAGTG	CGCAGTAGACCTTGCCAACAT
hrt *c-MYC*	TGCCTCAAATTGGACTTGG	GATTGAAATTCTGTGTAACTGC
hrt *OCT4*	CCTCACTTCACTGCACTGTA	CAGGTTTTCTTTCCCTAGCT
hrt *SOX2*	CCCAGCAGACTTCACATGT	CCTCCCATTTCCCTCGTTTT
hrt *KLF4*	GATGAACTGACCAGGCACTA	GTGGGTCATATCCACTGTCT
hrt *NANOG*	TGAACCTCAGCTACAAACAG	TGGTGGTAGGAAGAGTAAAG
hrt *GAPDH*	CAAGGTCATCCATGACAACTTTG	GTCCACCACCCTGTTGCTGTAG
hrt *AFP*	GAATGCTGCAAACTGACCACGCTGCAAC	TGGCATTCAAGAGGGTTTTCAGTCTGGA
hrt *SOX17*	CGCTTTCATGGTGTGGGCTAAGGACG	TAGTTGGGGTGGTCCTGCATGTGCTG
hrt *MSX1*	CGAGAGGACCCCGTGGATGCAGAG	GGCGGCCATCTTCAGCTTCTCCAG
hrt *BRACHYURY*	GCCCTCTCCCTCCCCTCCACGCACAG	CGGCGCCGTTGCTCACAGACCACAGG
hrt *MAP2*	CAGGTGGCGGACGTGTGAAAATTGAGA	CACGCTGGATCTGCCTGGGGACTGTG
hrt *PAX6*	CGGAGTGAATCAGCTCGGTG	CCGCTTATACTGGGCTATTTTGC
hrt *RAX*	GAATCTCGAAATCTCAGCCC	CTTCACTAATTTGCTCAGGAC
hrt *BRN3a*	GGGCAAGAGCCATCCTTTCAA	CTGTTCATCGTGTGGTACGTG
hrt *NEFM*	GAAATCGCTGCGTACAGAAAAC	TAATGGCTGTCAGGGCCTCTT

'hrt' means that human real - time PCR primers.

Supplement table S3: KEY RESOURCES TABLE

REAGENT or RESOURCE	SOURCE	IDENTIFIER
Antibodies		
SSEA4	Abcam	Cat# ab16287
OCT4	Abcam	Cat# ab19857
TRA-1-60	Abcam	Cat# ab16288
NANOG	Abcam	Cat# ab21624
SOX2	Abcam	Cat# ab97959
PAX6	Sigma	Cat# HPA030775
RAX	Novas	Cat# H000.0062-M02
SOX2	Sigma	Cat# AB5603
NESTIN	Scuz	Cat# SC23927
TJP1	Abcam	Cat# ab276131
ISL1	Abcam	Cat# 109517
BRN3a	Scuz	Cat# SC390078
SNCG	Invitrogen	Cat# AHB0261
THY1	Scuz	Cat# SC53116
ATOH7	Sigma	Cat# SAB1410371
β III TUBULIN	Abcam	Cat# ab78078
β III TUBULIN	Abclone	Cat# A18132
Goat anti - mouse IgG H+L (Alexa Fluor 594)	Abcam	Cat# ab150116
Goat anti - rabbit IgG H+L (Alexa Fluor 488)	Abcam	Cat# ab150077
Goat anti-mouse IgG H+L (Alexa Fluor 488)	Abcam	Cat# ab150113
Goat anti-rabbit IgG H+L (Alexa Fluor 594)	Abcam	Cat# ab150080
Critical Commercial Assays		
Enhanced ATP Assay Kit	Beyotime	Cat# S0027
Apoptosis Inducer Kit	Beyotime	Cat# C0006S
Caspase-3 Activity Assay Kit	Beyotime	Cat# C1116
MitoSOX™ Red Mitochondrial Superoxide Indicator, for live-cell imaging	Invitrogen	Cat# M36008
MitoTracker™ RedCMXRos Kit (Flow Cytometry)	Invitrogen	Cat# M7512
TRIzol reagent	Invitrogen	Cat# 15596018
PrimeScript II 1st strand cDNA synthesis Kit	TaKaRa	Cat# 6210A
MitoProbe™ TMRM Assay Kit for Flow	Invitrogen	Cat# M20036

Cytometry		
Human Dermal Fibroblast NucleofectorTM Kit	LONZA	Cat# VPD-1001
**Plasmids**		
pCXLE-EGFP	Addgene	Cat# 27082
pCXLE-hSK	Sigma	Cat# 27078
pCXLE-hUL	Sigma	Cat# 27080
pCXLE-hOCT3/4-shp53-F	Sigma	Cat# 27077
pCXWB-EBNA1	Promega	Cat# 37624
**Software**		
FIJI IMAGEJ	Image J	N/A
GraphPad Prism8	GraphPad Software	
Microsoft-Excel	Microsoft	N/A
7900 Real-Time PCR Software	Applied Biosystems	N/A
Olympus Fluoview Ver.4.0a Viewer	Olympus	N/A
Stats	IBM	N/A
